# Diagnostic Sequences That Distinguish *M. avium* Subspecies Strains

**DOI:** 10.3389/fvets.2020.620094

**Published:** 2021-01-28

**Authors:** John P. Bannantine, Judith R. Stabel, Darrell O. Bayles, Cyril Conde, Franck Biet

**Affiliations:** ^1^USDA-Agricultural Research Service, National Animal Disease Center, Ames, IA, United States; ^2^INRAE, Université de Tours, ISP, Nouzilly, France

**Keywords:** *M. avium*, *Mycobacterium*, paratuberculosis (*Map*), whole genome comparison, diagnostics, PCR

## Abstract

Over a decade ago *Mycobacterium avium* subspecies *paratuberculosis* (*Map*) specific genes were initially identified in a whole genome context by comparing draft genome sequences of *Map* strain K-10 with *Mycobacterium avium* subspecies *hominissuis* (*Mah*) strain 104. This resulted in identification of 32 *Map* specific genes, not including repetitive elements, based on the two-genome comparison. The goal of this study was to define a more complete catalog of *M. avium* subspecies-specific genes. This is important for obtaining additional diagnostic targets for Johne's disease detection and for understanding the unique biology, evolution and niche adaptation of these organisms. There are now over 28 complete genome sequences representing three *M. avium* subspecies, including *avium* (*Maa*), *Mah*, and *Map*. We have conducted a comprehensive comparison of these genomes using two independent pan genomic comparison tools, PanOCT and Roary. This has led to the identification of more than 250 subspecies defining genes common to both analyses. The majority of these genes are arranged in clusters called genomic islands. We further reduced the number of diagnostic targets by excluding sequences having high BLAST similarity to other mycobacterial species recently added to the National Center for Biotechnology Information database. Genes identified as diagnostic following these bioinformatic approaches were further tested by DNA amplification PCR on an additional 20 *M. avium* subspecies strains. This combined approach confirmed 86 genes as *Map*-specific, seven as *Maa*-specific and three as *Mah*-specific. A single-tube PCR reaction was conducted as a proof of concept method to quickly distinguish *M. avium* subspecies strains. With these novel data, researchers can classify isolates in their freezers, quickly characterize clinical samples, and functionally analyze these unique genes.

## Introduction

Accurate diagnosis of mycobacterial infections is important for effective disease management within the livestock production industry. National and state veterinary laboratories are routinely confronted with unresolved cases of non-tuberculous mycobacterial (NTM) infections that are not further delineated due to complicated cross-reactive results as well as a lack of time and funds. However, correct identification of *M. avium* subspecies is very important because of the divergent, yet prominent, clinical relevance that exists among these strains (1). The *Mycobacterium avium* subspecies comprise three predominant, closely-related subspecies, *M. avium* subspecies *avium* (*Maa*), *M. avium* subspecies *hominissuis* (*Mah*), and *M. avium* subspecies *paratuberculosis* (*Map*) and their genetic similarity has limited the identification of diagnostic targets that distinguish each subspecies. A forth subspecies has been reported (*silvaticum*), but the lack of circulating strains and sequences in public databases suggests this subspecies designation may be artifactual (2). *Map* is the causative agent of Johne's disease, which is a chronic intestinal ailment of ruminants that results in significant economic loss to dairy, meat and wool industries (3, 4). *Mah* is an opportunistic pathogen of swine and humans that manifests most commonly as a pulmonary infection in humans (5), while *Maa* is primarily a pathogen of birds (6).

In this report, the terms *Map* and non-*Map* are used to divide the *M. avium* subspecies. This is due to the focus on diagnostic *Map* sequences, which are major tools for research and surveillance of Johne's disease. Conversely, very few studies have reported sequences that are diagnostic for non-*Map* subspecies. The only known sequences that are specific for the *Maa* and *Mah* subspecies include IS1245 (7) and genes contained within insertions or other genetic rearrangements termed large sequence polymorphisms (LSPs) (8). Whole genome comparative methods are a comprehensive approach for identifying specific genes useful in molecular diagnostic tests. In this approach, genomes from closely related organisms are compared to identify genes uniquely correlated with particular groups. A complicating factor is that *M. avium* subspecies strains studied thus far all share >98% average nucleotide identity across the entire genome (2). In addition, the core genome appears stable for *Map* with 80% of the genome sequence comprising genes present in all strains, whereas the core genomes of non-*Map* subspecies comprise only 40% of genes (2). Despite strong genetic similarity and availability of commonly used diagnostic targets (i.e., IS900), there are still undiscovered and unexplored genetic targets that may be used in diagnostic tests to distinguish the *M. avium* subspecies. These sequences are also important to understanding the unique biology, evolution and niche adaptation of these strains. A primary goal of this study is to identify genetic targets that can distinguish *M. avium* subspecies from each other.

Despite high sequence identity among these subspecies, some specific sequences have long been identified and used for diagnostics of Johne's disease. The most commonly used target is the repetitive sequence, IS900, which is present in 16–22 copies in *Map*. This insertion element was identified as *Map*-specific long before whole-genome sequences became available (9, 10). Other sequences reported as specific to *Map* include the single-copy genes *hspX* (MAP_RS11095; MAP2182c) (11, 12), F57 (MAP_RS22535; MAP_0865) (13), and locus 251 (MAP_RS14130; MAP2765c) (14, 15). Locus 251 was originally identified from shotgun sequence data shortly before the first complete genome of an *M. avium* subspecies was assembled (15) and later used as a target in commercial PCR assays. F57 was originally identified from a genomic DNA library screen (16) and was later discovered as part of the MAP_0865 coding sequence present on a large sequence polymorphism termed LSP4 (17). This gene has also been a common target in real time PCR assays (13, 18, 19). Thirty-two *Map*-specific genes were identified by genomic hybridizations to DNA microarrays (20–22), but this approach lacked the resolution and completeness of whole-genome sequences.

To identify all remaining sequences specific for *Map* and new diagnostic sequences for *Maa* and *Mah*, complete genomes from several strains were compared and candidate genes identified. Two independent bioinformatic analyses were first conducted to obtain subspecies specific genes in *M. avium*. These analyses included Roary (23) and PanOCT (24) pan genome tools, which identify core and accessory genes for a group of closely related organisms. PanOCT works by joining homologous and conserved gene neighborhoods to cluster orthologous proteins while Roary preclusters highly similar protein sequences before BLASTP similarity searching. PanOCT analysis yields a variety of comparative data about the input genomes (24), but one of the more useful outputs are the match tables. These tables list orthologous clusters of genes along with information on the cutoff metrics that would result in genes being declared absent in the match table. Match tables were constructed from PanOCT analysis showing all genomes, including their corresponding genes and percent identity of their gene products. These tools were used previously to determine the core genome of *Map* and non-*Map* strains (2). Finally, once specific genes are identified, DNA amplification primers can then be designed for further testing on a panel of organisms representing additional *M. avium* strains.

## Materials and Methods

### *In silico* Identification of Subspecies Specific Genes

PanOCT output includes a match table of all genes in every input genome (Genbank flatfile format) with orthologs arranged in rows and genomes arranged in columns. The input genomes included the 29 *M. avium* genomes reported in Tables 1, 2 of (2). When orthologs are not present in a genome(s), this is interpreted as one of several possibilities: the amino acid percent identity is <35%, the BLAST E-value was >1 × 10^−5^, the minimum percent match length of subject and query (default is 1%) was not met, the frame-shift overlap parameter (default is 1.33) triggered a non-match, there were more than 20 amino acids at the beginning or end of a match that were missing, and finally, there was not at least one match that confirmed a protein fragment/frame-shift. Roary (v3.11.2) was used to define the accessory genome of each subspecies and was launched with –e and –n options to compute rapid core gene alignment. Coding sequences from both analyses were assembled and compared. Only subspecies-specific genes identified by both approaches were analyzed by DNA amplification.

**Table 1:** Genomic DNA templates used in DNA amplification experiments.

**Species**	**Subspecies**	**Strain**	**Culture no**.	**Host**	**Notes**
*Avium*	*Paratuberculosis*	K10	6121	Cow	
*Avium*	*Paratuberculosis*	Linda	6100	Human	
*Avium*	*Paratuberculosis*	ATCC 19698-1974	6002	Cow	
*Avium*	*Paratuberculosis*	Isolate 4025		Cow	Isolated from the jejunum of a Guernsey cow
*Avium*	*Paratuberculosis*	Sheep 407	6096	Sheep	Distal ileum isolate from a Suffolk in the US
*Avium*	*Paratuberculosis*	Bovine isolate	6013	Cow	From a high shedding cow by Dr. Whitlock, U Penn
*Avium*	*Paratuberculosis*	Bovine isolate	5077	Cow	From the IC valve of a Holstein cow in 1993
*Avium*	*Paratuberculosis*	117p	6116	Goat	Isolate from the Veterinary institute of Norway
*Avium*	*Paratuberculosis*	S397	6101	Sheep	A sheep isolate in the US
*Avium*	*Hominissuis*	H.S. 09-5894	6138	Cow	
*Avium*	*Hominissuis*	H.S. 10-1725	6140	Cow	
*Avium*	*Hominissuis*	MA 1115	6106		
*Avium*	*Hominissuis*	H.S. 10-05561	6124	Pig	Swine isolate in 2008
*Avium*	*Hominissuis*	H.S. 10-1519	6139	Cow	
*Avium*	*Avium*	ATCC 35719	6004	Chicken	MAIS complex 2
*Avium*	*Avium*	wood pigeon A	6007	Bird	
*Avium*	*Avium*	Strain 18	6005		Former vaccine strain
*Avium*	*Avium*		6084	Rabbit	Pygmy rabbit isolate
*Intracellulare*		ATCC 35773	6010	Swine	MAIS complex 6

**Table 2:** Number of *M. avium* subspecies specific genes identified by each analysis.

**Subspecies (*n*)**	**PanOCT**	**Roary**	**Both**
*Maa* (2)	158	213	157
*Mah* (13)	9	13	8
*Map* (13)	115	155	114

### Primer Design

Primers were designed using the National Center for Biotechnology Information (NCBI) primer search software (https://www.ncbi.nlm.nih.gov/tools/primer-blast/). Parameters that differed from the defaults included minimum product size of 300 bp, maximum product size of 800 bp, optimal primer melting temperature of 68°C with a max melting temperature of 70°C, and database selection was RefSeq genomes. At least 10 candidate primer pairs obtained from NCBI primer searches were tested on each gene *in silico* using MacVector version 17.5. Primer pairs were discarded if any bound within the product or if they formed hairpin and self-duplex structures. Only one set of primers was kept for each gene to use in DNA amplification studies. Accepted primer pairs are shown in Supplementary Table 1 for all genes analyzed. If the open reading frames were small and adjacent to each other, a forward primer was designed in one coding sequence and the reverse primer was designed in the neighboring coding sequence. This approach provided more sequence to design optimal primer pairs and lessened the number of PCRs to be conducted. In one case, a primer pair was designed that spanned three small coding sequences, MAP_RS10970, MAP_RS10975, and MAP_RS10980.

### Functional Analysis of Genes

All Roary *Map*-specific protein sequences of each gene (155 total) were extracted using an in-house python script (Supplementary Materials) based on Biopython (https://biopython.org). KEGG orthologs for each protein sequence were assigned using kofamscan tool (https://github.com/takaram/kofam_scan) based on HMM profile search with default parameters. Kofamscan results were parsed using another in-house python script (searchKEGG.py). Briefly, for each gene, we selected the best result based either on the ratio value compute as score / threshold_score where available or on the lower e-value. K numbers were then used to retrieve the complete KEGG hierarchies and functional classes were assigned based on the KEGG hierarchies. For functional analysis using eggNOG, all Roary *Map*-specific protein sequences were again extracted. Proteins were aligned against the eggNOG database v5.0 using Diamond (https://github.com/bbuchfink/diamond) and functional annotation was assigned to each protein sequence when available using emapper.py python script from eggnog-Mapper tool. Results were parsed using an in-house python script (Supplementary Materials) in order to extract relevant information.

### DNA Amplification

Genomic DNA was purified from mycobacterial strains shown in Table 1 using the method described previously (15). Advantage GC-2 (Takara Bio) was used for all amplifications on 100 ng of DNA and 100 μM of each primer. All reactions were repeated twice and if an unexpected result occurred, the reaction was again tested using EconoTaq PLUS GREEN master mix (Lucigen). All amplification reactions were conducted on an Applied Biosystems GeneAmp PCR System 9700 as follows: a single denaturation step at 94°C for 8 min; then 25 cycles of 94°C for 30 s, 70°C for 1 min, 72°C for 1 min; then a final extension at 72°C for 5 min. The single reaction multiplex PCRs were performed using the same conditions above, except three sets of primers (highlighted red in Supplementary Table 1) were included in each reaction. *In silico* PCR was performed with MacVector 17.5 using the primer test feature and the same primers listed in Supplementary Table 1. DNA templates for *in silico* PCR consisted of complete genome sequences of *Mycobacterium* available in public sequence databases.

### Data Availability

Raw sequence data supporting the results of this article were deposited in the European Nucleotide Archive (ENA) and NCBI under accession PRJEB2204.

## Results

### Bioinformatic Analysis of *M. avium* Subspecies-Specific Genes

#### Map

The initial number of subspecies-specific gene candidates were obtained by two comparative genomic methods, Roary and PanOCT analysis. The same set of 28 complete genomes that were used in our previous study (2) was included herein. PanOCT analysis revealed a total of 115 genes that are present in all *Map* genomes, but absent in all non-*Map* genomes while Roary analysis identified 155 *Map*-specific genes (Table 2; Supplementary Table 2). The total number of *Map*-specific genes identified by either Roary or PanOCT analysis was 156 (Supplementary Table 2).

Of the 156 total genes, Roary analysis uncovered 41 genes not found by PanOCT analysis whereas one gene was identified by PanOCT analysis that was not listed by Roary. The reason for this single-gene discrepancy is due to different translational start sites in one of the 13 *Map* orthologs (EGA31_RS21685 in the Telford strain) encoding an alpha/beta hydrolase, which made this gene appear as though it was not present in all *Map* strains analyzed (Supplementary Figure 1; Table 3). Thus, it was excluded by Roary analysis. The 41 genes present by Roary analysis but absent by PanOCT is due to the different clustering methods used by these bioinformatic tools. However, it is of interest to further explore this subset of 41 genes to understand which bioinformatic approach might be better for defining these gene targets. BLAST analysis of these 41 genes against the NCBI database showed reasonably strong homologies to other, more distantly related, *Mycobacterium* species (Supplementary Table 3). These results suggest that PanOCT may be better suited to consider more evolutionarily distant strains than Roary. Nonetheless, 114 *Map*-specific gene candidates are present in both analyses (Supplementary Table 2). This result excludes *Map*-specific repetitive sequences, such as IS900, which are not annotated in the E1 and E93 genome sequences (2).

**Table 3:** *M. avium* subspecies-specific gene discrepancy in Roary analysis.

**Subspecies**	**Strain**	**Locus tag**	**Description**	**Amino acids**	**Roary**
*paratuberculosis*	K-10	MAP_RS19250	Alpha/beta hydrolase	MAVSATAGI…	Yes
*paratuberculosis*	MAP4	MAP4_RS00065	Alpha/beta hydrolase	MAVSATAGI…	Yes
*paratuberculosis*	E1	RC58_RS00065	Alpha/beta hydrolase	MAVSATAGI…	Yes
*paratuberculosis*	E93	RE97_RS00065	Alpha/beta hydrolase	MAVSATAGI…	Yes
*paratuberculosis*	India 2008	A0V42_RS19260	Alpha/beta hydrolase	MAVSATAGI…	Yes
*paratuberculosis*	FDAARGOS	CEP84_RS06855	Alpha/beta hydrolase	MAVSATAGI…	Yes
*paratuberculosis*	JII-1961	CEG92_RS19670	Alpha/beta hydrolase	MAVSATAGI…	Yes
*paratuberculosis*	MAPK_CN7/15	EC391_RS14825	Alpha/beta hydrolase	MAVSATAGI…	Yes
*paratuberculosis*	MAPK_CN9/15	EC390_RS07915	Alpha/beta hydrolase	MAVSATAGI…	Yes
*paratuberculosis*	MAPK_CN4/13	EGM63_RS09965	Alpha/beta hydrolase	MAVSATAGI…	Yes
*paratuberculosis*	MAPK_JB16/15	EGM64_RS22685	Alpha/beta hydrolase	MAVSATAGI…	Yes
*paratuberculosis*	MAPK_JJ1/13	EGM60_RS19585	Alpha/beta hydrolase	MAVSATAGI…	Yes
*paratuberculosis*	Telford	EGA31_RS21685	Alpha/beta hydrolase	MLLRASRYF…	No
*hominissuis*	104	MAV_RS08635	Hydroxycinnamic acid hydroxylase	MTDSPAYK…	Yes
*hominissuis*	TH135	MAH_RS07880	Propionate hydroxylase	MPVVIVGAG…	No
*hominissuis*	OCU464	KV38_RS08390	Hydroxycinnamic acid hydroxylase	MTDSPAYK…	Yes
*hominissuis*	H87	BS641_RS14715	Propionate hydroxylase	MTDSPAYK…	Yes
*hominissuis*	HP17	BEP52_RS08480	Hydroxycinnamic acid hydroxylase	MPVVIVGAG…	No
*hominissuis*	OCU901_S2_2s	BJP78_RS08465	Hydroxycinnamic acid hydroxylase	MPVVIVGAG…	No
*hominissuis*	OCU873s_P7_4s	BJP74_RS08005	Hydroxycinnamic acid hydroxylase	MPVVIVGAG…	No
*hominissuis*	MAC109	DFS55_RS16395	Hydroxycinnamic acid hydroxylase	MTDSPAYK…	Yes
*hominissuis*	mc2 2500	EX350_RS14220	Hydroxycinnamic acid hydroxylase	MTDSPAYK…	Yes
*hominissuis*	101174	FCV17_RS18625	Hydroxycinnamic acid hydroxylase	MTDSPAYK…	Yes
*hominissuis*	101034	FCV16_RS11165	Hydroxycinnamic acid hydroxylase	MTDSPAYK…	Yes
*hominissuis*	101115	FCV18_RS02655	Hydroxycinnamic acid hydroxylase	MTDSPAYK…	Yes
*hominissuis*	JP-H-1	JPH1_RS10225	Hydroxycinnamic acid hydroxylase	MTDSPAYK…	Yes

*Genes listed in the above table are all orthologs in PanOCT, but those in red font have different translational starts. All the sequences in red font also indicate they are not listed in the Roary analysis*.

*This translational start for Telford was downstream of that for the rest of the Map genomes*.

#### Non-map

The *Mah*-specific and *Maa*-specific genes were also obtained using similar analyses. There are only nine *Mah*-specific genes identified by PanOCT and 13 by Roary analysis (Table 2; Supplementary Table 4). Eight of these genes are present in both analyses. One gene, encoding hydroxycinnamic acid hydroxylase (Table 3), that is uniquely present in the PanOCT analysis was due to differences in the clustering of orthologous genes. In this case, the BLAST score ratio, when used alone, would not classify these orthologs as a cluster. Therefore, Roary excluded it. However, when the conserved gene neighborhood is factored in the PanOCT analysis, these genes are found rooted in the same source. This might be what one would expect if there was a single event that introduced an entire cluster followed by divergence of a gene within that cluster. Conversely, there are five *Mah* genes identified as specific only by Roary analysis. As before, these differences were due to the clustering methods used by these bioinformatic tools (see Discussion). The largest set of subspecies-specific genes were obtained for *Maa* strains, which included 157 genes identified by both analyses (Table 2; Supplementary Table 5).

#### Location of *M. avium* Subspecies-Specific Genes

**Figure 1:**
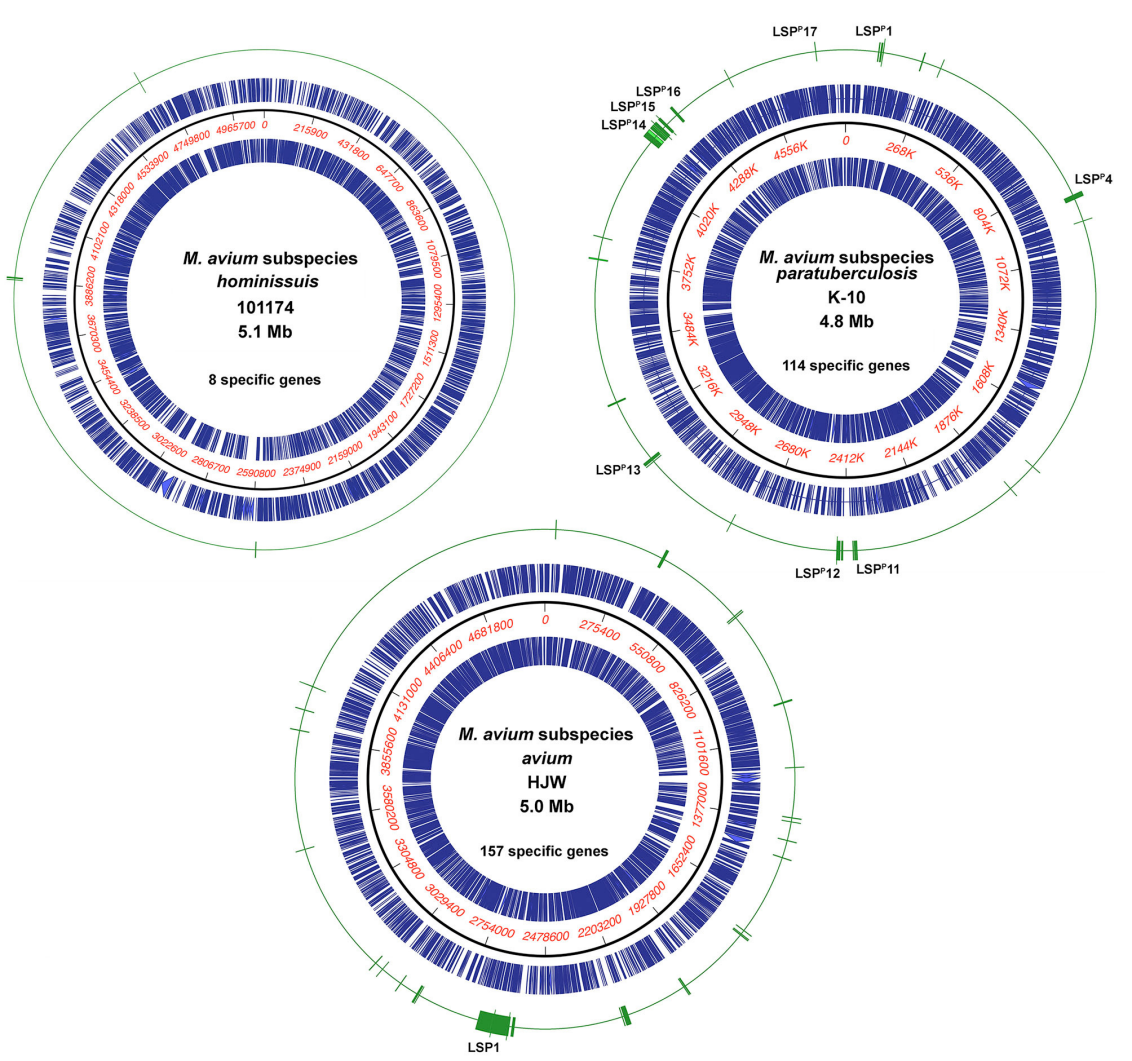
Location of subspecies specific genes on representative *M. avium* genome maps. Representative genomes from each of the three subspecies are shown with the concentric circles from outer most ring to inner most ring as follows: subspecies-specific genes (green), coding sequences forward strand (blue), nucleotide scale (black line with red numbers), coding sequences reverse strand (blue). All subspecies-specific genes identified by both PanOCT and Roary analyses are shown. The strain and genome size are indicated in the center of the circle along with the number of specific genes for that subspecies. Genome maps were drawn using MacVector 17.5. *Map* LSPs that correspond to those described by Semret et al. (17) are shown as well as LSP1 in *Mah* described by Semret et al. (8).

Subspecies specific genes are not randomly distributed over the entire genome but tend to be organized in clusters, termed large sequence polymorphisms (LSPs) (8, 17). This fact is supported by the results of this study, where several specific genes are contained within LSPs regardless of the subspecies (Figure 1). *Maa* shows the most clusters (26 total), followed by *Map* and then *Mah*, which only has four clusters of eight total genes. For example, the largest genomic island is just over 100 kb in *Maa* and it contains 97 *Maa*-specific genes. This island was previously described as LSP1 by Semret et al. (8). The location of LSP1 is at 7 o'clock on the *Maa* genome map in Figure 1. Likewise, the largest genomic island in *Map* includes 30 genes spanning 55 kb and was previously named LSP^P^14 (17). Not all genes within any defined LSP were found specific to a given subspecies in this study.

#### BLAST Similarity and *in silico* PCR Analysis

The 114 *Map*-specific genes were next analyzed by similarity searches against other complete mycobacterial genomes including members of the *M. tuberculosis* complex and non-tuberculosis mycobacteria closely related to the MAC complex (25, 26). Several *Map*-specific genes showed surprisingly strong similarity to mycobacterial genomes that were recently added to public sequence databases (Supplementary Table 6). These include *M. marseillense, M. lepraemurium, M. branderi*, and *Mycolicibacterium doricum*. *In silico* PCR using primers designed for this study (Supplementary Table 1) were tested on the strongest matching genome sequences obtained from BLAST analysis and amplification products of the correct size were obtained for nine *Map* genes (Supplementary Table 6). Removing these nine targets reduced the list of *Map* genes to 104 that are defined as *Map*-specific following PanOCT, Roary and *in silico* PCR analysis. Within that group of 104 genes, 54 were highly specific to *Map* as they had no matches in the NCBI sequence database (indicated by “no result” in Supplementary Table 6). Two additional *M. avium* genomes, *Map* DSM 44135 (27) and *Maa* ATCC 25291 (28), were published after this study began and *in silico* PCR on those genome sequences shows agreement with subspecies specificity for all genes except FCV17_RS22495, a *Mah* gene that yielded a correct size product in *Maa* ATCC 25291 (Supplementary Table 4).

#### Gene Ontology of Map-Specific Genes

**Figure 2:**
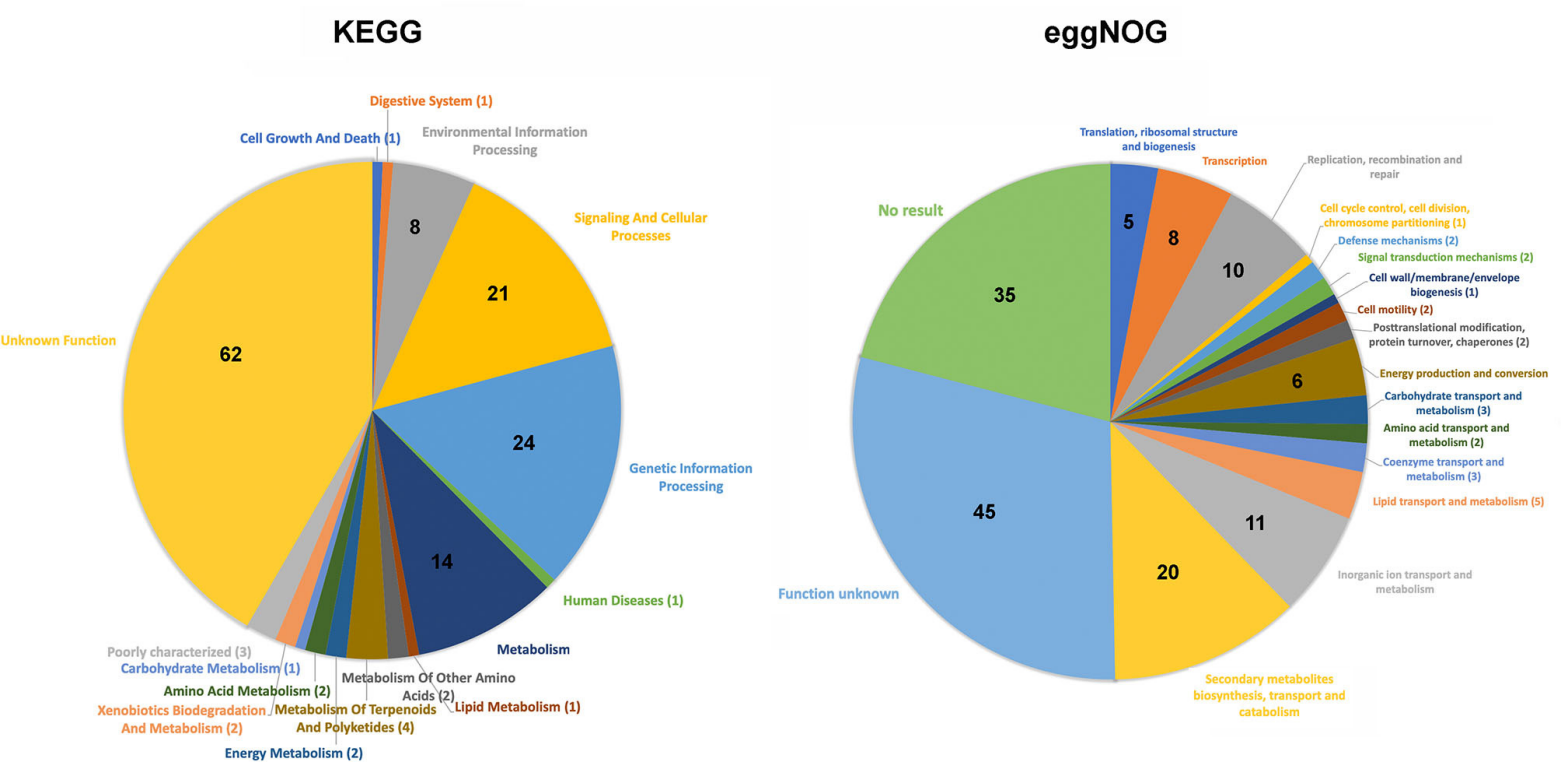
Pie chart distribution of the functional categories obtained for *Map*-specific genes via KEGG (left chart) and eggnog analysis (right chart). The number of *Map*-specific genes present in each defined category are indicated. Note that in both analyses the unknown function category contains the largest percentage of genes.

Functional analysis was performed on *Map*-specific genes using Kyoto Encyclopedia of Genes and Genomes (KEGG) (29) and evolutionary genealogy of genes: Non-supervised Orthologous Groups (eggNOG) (30). These genes were separated into 16 functional classifications by KEGG and 18 by eggNOG (Figure 2). Other than the most predominant unknown function class, KEGG analysis revealed 14 lipid transport system proteins, which likely remodel the cell wall in some way. EggNOG analysis showed the presence of Mce family proteins (n=6), and ABC transporters (n=5), but were predominantly genes of unknown function (n=45). The high number of genes with no assigned function suggests there are a lot of unknowns about what makes *Map* unique to other bacteria.

### DNA Amplification of Subspecies Specific Genes

*Taq* polymerase-based DNA amplification of selected genes was performed on purified genomic DNA from 16 additional *M. avium* subspecies strains and *Map* K-10 as a control. All 104 *Map*-specific genes (Supplementary Table 2) and all eight *Mah*-specific genes (Supplementary Table 4) were tested. In addition, a subset of eight genes from the 157 *Maa*-specific genes (Supplementary Table 5) were also tested by DNA amplification. IS900 and IS1245 repetitive element targets were included as controls. The results show that 106 of 120 genes tested were confirmed as subspecies-specific (Table 4). A gene was dropped from the subspecies-specific gene list if it failed to amplify in any target strains or if it did amplify in any non-target strains. Such unexpected reactions are highlighted in yellow (if negative) and green (if positive) in Table 4.

**Table 4:** DNA amplification of purified genomic DNA from additional *M. avium* subspecies strains.

	**PCR product**	***M. avium*** **subspecies** ***paratuberculosis***								***M. avium*** **subspecies** ***hominissuis***				***M. avium*** **subspecies** ***avium***			***M. intracellulare***
**Locus tag**	**Length (bp)**	**K-10**	**Linda**	**ATCC19698**	**4025**	**Bovine str**.	**Goat-bov**	**Sheep 407**	**S397**	**2 strains**	**10-1519**	**10-1725**	**6084**	**6106/6007**	**ATCC35719**	**Strain 18**	**ATCC35773**
MAP_RS00485-MAP_RS00490	520	+	–	+	+	+	+	+	+	–	–	–	–	–	–	–	–
MAP_RS00515-MAP_RS00520	307	+	+	+	+	+	+	+	+	–	+	–	+	–	–	–	–
MAP_RS22390-MAP_RS00525	560	+	+	+	+	+	+	+	+	–	+	–	+	–	–	–	–
MAP_RS00540	300	+	+	+	+	+	+	+	+	–	–	–	–	–	–	–	–
MAP_RS00545	458	+	+	+	+	+	+	+	+	–	–	–	–	–	–	–	–
MAP_RS01130-MAP_RS01135	365	+	+	+	+	+	+	+	+	–	–	–	–	–	–	–	+
MAP_RS04345-MAP_RS04350	301	+	+	+	+	+	+	+	+	–	–	–	–	–	–	–	–
MAP_RS04355	302	+	+	+	+	+	+	+	+	–	–	–	–	–	–	–	–
MAP_RS04705	300	+	+	+	+	+	+	+	+	–	–	–	–	–	–	–	–
MAP_RS08655	334	+	+	+	+	+	+	+	+	–	+	–	+	+	–	+	+
MAP_RS10920	319	+	+	+	+	+	+	+	+	–	–	–	–	–	–	–	–
MAP_RS10925-MAP_RS10930	353	+	–	+	+	+	+	+	+	–	–	–	–	–	–	–	–
MAP_RS10940	312	+	+	+	+	+	+	+	+	–	–	–	–	–	–	–	–
MAP_RS10945	355	+	+	+	+	+	+	+	+	–	–	–	–	–	–	–	–
MAP_RS10965	364	+	+	+	+	+	+	+	+	–	–	–	–	–	–	–	–
MAP_RS10970-MAP_RS10980	353	+	–	+	+	+	+	+	+	–	–	–	–	–	–	–	–
MAP_RS11080	300	+	+	+	+	+	+	+	+	–	–	–	–	–	–	–	–
MAP_RS11085	301	+	+	+	+	+	+	+	+	–	–	–	–	–	–	–	–
MAP_RS11090	384	+	+	+	+	+	+	+	+	–	–	–	–	–	–	–	–
MAP_RS11095	336	+	+	+	+	+	+	+	+	–	–	–	–	–	–	–	–
MAP_RS11100	372	+	+	+	+	+	+	+	+	–	–	–	–	–	–	–	–
MAP_RS11105	328	+	+	+	+	+	+	+	+	–	–	–	–	–	–	–	–
MAP_RS11130	527	+	+	+	+	+	+	+	+	–	–	–	–	–	–	–	–
MAP_RS11135	402	+	+	+	+	+	+	+	+	–	–	–	–	–	–	–	–
MAP_RS11140	403	+	+	+	+	+	+	+	+	–	–	–	–	–	–	–	–
MAP_RS11155	628	+	–	+	+	+	+	+	+	–	–	–	–	–	–	–	–
MAP_RS11160	309	+	+	+	+	+	+	+	+	–	–	–	–	–	–	–	–
MAP_RS11165-MAP_RS11170	428	+	+	+	+	+	+	+	+	–	–	–	–	–	–	–	–
MAP_RS12615	300	+	+	+	+	+	+	+	+	–	–	–	–	–	–	–	–
MAP_RS14055	180	+	+	+	+	+	+	+	+	–	–	–	–	–	–	–	–
MAP_RS14060	301	+	+	+	+	+	+	+	+	–	–	–	–	–	–	–	–
MAP_RS14070	191	+	+	+	+	+	+	+	+	–	–	–	–	–	–	–	–
MAP_RS14095	231	–	–	–	–	–	–	–	–	–	–	–	–	–	–	–	–
MAP_RS14110	272	+	+	+	+	+	+	+	+	–	–	–	–	–	–	–	–
MAP_RS14120	225	+	+	+	+	+	+	+	+	–	–	–	–	–	–	–	–
MAP_RS14130	381	+	+	+	+	+	+	+	+	–	–	–	–	–	–	–	–
MAP_RS14135	211	+	+	+	+	+	+	+	+	–	–	–	–	–	–	–	–
MAP_RS14140	489	+	+	+	+	+	+	+	+	–	–	–	–	–	–	–	–
MAP_RS15170	419	+	–	+	+	+	+	+	+	–	–	–	–	–	–	–	–
MAP_RS17665	380	+	+	+	+	+	+	+	+	–	–	–	–	–	–	–	–
MAP_RS17670	264	+	+	+	+	+	+	+	+	–	–	–	–	–	–	–	–
MAP_RS19105	333	+	+	+	+	+	+	+	+	–	–	–	–	–	–	–	–
MAP_RS19110	333	+	+	+	+	+	+	+	+	–	–	–	–	–	–	–	–
MAP_RS19115	353	+	+	+	+	+	+	+	+	–	–	–	–	–	–	–	–
MAP_RS19120	300	+	+	+	+	+	+	+	+	–	–	–	–	–	–	–	–
MAP_RS19130	333	+	+	+	+	+	+	+	+	–	–	–	–	–	–	–	–
MAP_RS19135	316	+	+	+	+	+	+	+	+	–	–	–	–	–	–	–	–
MAP_RS19140	310	+	+	+	+	+	+	+	+	–	–	–	–	–	–	–	–
MAP_RS19145	368	+	+	+	+	+	+	+	+	–	–	–	–	–	–	–	–
MAP_RS19150	760	+	+	+	+	+	+	+	+	–	–	–	–	–	–	–	–
MAP_RS19155	340	+	+	+	+	+	+	+	+	–	–	–	–	–	–	–	–
MAP_RS19160	426	+	+	+	+	+	+	+	+	–	–	–	–	–	–	–	–
MAP_RS19165	406	+	–	+	+	+	+	+	+	–	–	–	–	–	–	–	–
MAP_RS19170	620	+	+	+	+	+	+	+	+	–	–	–	–	–	–	–	–
MAP_RS19185	597	+	+	+	+	+	+	+	+	–	–	–	–	–	–	–	–
MAP_RS19190	616	+	+	+	+	+	+	+	+	–	–	–	–	–	–	–	–
MAP_RS19195	470	+	+	+	+	+	+	+	+	–	–	–	–	–	–	–	–
MAP_RS19200	308	+	+	+	+	+	+	+	+	–	–	–	–	–	–	–	–
MAP_RS19205	492	+	+	+	+	+	+	+	+	–	–	–	–	–	–	–	–
MAP_RS19215	340	+	+	+	+	+	+	+	+	–	–	–	–	–	–	–	–
MAP_RS19220	342	+	+	+	+	+	+	+	+	–	–	–	–	–	–	–	–
MAP_RS19225	316	+	+	+	+	+	+	+	+	–	–	–	–	–	–	–	–
MAP_RS19230	602	+	+	+	+	+	+	+	+	–	–	–	–	–	–	–	–
MAP_RS19240-MAP_RS19245	326	+	+	+	+	+	+	+	+	–	–	–	–	–	–	–	–
MAP_RS19255	331	+	+	+	+	+	+	+	+	–	–	–	–	–	–	–	–
MAP_RS19265	328	+	+	+	+	+	+	+	+	–	–	–	–	–	–	–	–
MAP_RS19270	445	+	+	+	+	+	+	+	+	–	–	–	–	–	–	–	–
MAP_RS19275	364	+	+	+	+	+	+	+	+	–	–	–	–	–	–	–	–
MAP_RS19280	462	+	+	+	+	+	+	+	+	–	–	–	–	–	–	–	–
MAP_RS19305-MAP_RS19310	323	+	+	+	+	+	+	+	+	–	–	–	–	–	–	–	–
MAP_RS19325-MAP_RS19330	356	+	+	+	+	+	+	+	+	–	–	–	–	–	–	–	–
MAP_RS19335	380	+	+	+	+	+	+	+	+	–	–	–	–	–	–	–	–
MAP_RS19345	352	+	+	+	+	+	+	+	+	–	–	–	–	–	–	–	–
MAP_RS19350	335	+	+	+	+	+	+	+	+	–	–	–	–	–	–	–	–
MAP_RS19355	351	+	+	+	+	+	+	+	+	–	–	–	–	–	–	–	–
MAP_RS19565	343	+	+	+	+	+	+	+	+	–	–	–	–	–	–	–	–
MAP_RS19570	150	+	+	+	+	+	+	+	+	–	–	–	–	–	–	–	–
MAP_RS19575	458	+	+	+	+	+	+	+	+	–	–	–	–	–	–	–	–
MAP_RS19580	310	+	+	+	+	+	+	+	+	–	–	–	–	–	–	–	–
MAP_RS22425	322	+	+	+	+	+	+	+	+	–	–	–	–	–	–	–	–
MAP_RS22505	342	+	+	+	+	+	+	+	+	–	–	–	–	–	–	–	–
MAP_RS22515	420	+	+	+	+	+	+	+	+	–	–	–	–	–	–	–	–
MAP_RS22520	492	+	+	+	+	+	+	+	+	–	–	–	–	–	–	–	–
MAP_RS22525	371	+	+	+	+	+	+	+	+	–	–	–	–	–	–	–	–
MAP_RS22535	305	+	+	+	+	+	+	+	+	–	–	–	–	–	–	–	–
MAP_RS22540	221	+	+	+	+	+	+	+	+	–	–	–	–	–	–	–	–
MAP_RS22625	333	+	+	+	+	+	+	+	+	–	–	–	–	–	–	–	–
MAP_RS22710	338	+	+	+	+	+	+	+	+	–	–	–	–	–	–	–	–
MAP_RS22805	307	+	+	+	+	+	+	+	+	–	–	–	–	–	–	–	–
MAP_RS22810	314	+	+	+	+	+	+	+	+	–	–	–	–	–	–	–	–
MAP_RS22985	343	+	+	+	+	+	+	+	+	–	–	–	–	–	–	–	–
MAP_RS23005	327	+	+	+	+	+	+	+	+	–	–	–	–	–	–	–	–
IS900	229	+	+	+	+	+	+	+	+	–	–	–	–	–	–	–	–
FCV17_RS12965	377	–	–	–	–	–	–	–	–	+	+	+	+	–	–	–	+
FCV17_RS12970	411	–	–	–	–	–	–	–	–	+	+	+	+	–	–	–	+
FCV17_RS18600	315	–	–	–	–	–	–	+	–	+	+	+	+	–	–	–	+
FCV17_RS18630	463	–	–	–	–	–	–	–	–	+	+	+	+	–	–	–	–
FCV17_RS18635	481	–	–	–	–	–	–	–	–	+	+	+	+	–	–	–	+
FCV17_RS18640	309	–	–	–	–	–	–	–	–	+	+	+	+	–	–	–	–
FCV17_RS18645	316	–	–	–	–	–	–	–	–	+	+	+	+	–	–	–	–
FCV17_RS22495	184	–	–	–	–	–	–	–	–	+	+	–	+	+	+	+	+
DBO90_RS12280	366	–	–	–	–	–	–	–	–	–	–	–	–	+	+	+	–
DBO90_RS12340	402	–	–	–	–	–	–	–	–	–	–	–	–	+	+	+	–
DBO90_RS12410	330	–	–	–	–	–	–	–	–	–	–	–	–	+	+	+	–
DBO90_RS12500	308	–	–	–	–	–	–	–	–	–	–	–	–	+	+	+	–
DBO90_RS12630	368	–	–	–	–	–	–	–	–	–	–	–	–	+	+	+	–
DBO90_RS13535	469	+	–	–	+	–	+	–	–	–	–	–	–	+	+	+	–
DBO90_RS18520	358	–	–	–	–	–	–	–	–	–	–	–	–	+	+	+	–
DBO90_RS12105	310	–	–	–	–	–	–	–	–	–	–	–	–	+	+	+	–
IS1245	427	–	–	–	–	–	–	–	–	+	+	+	+	+	+	+	

*Amplification results with a green highlight indicate an unexpected positive reaction while results with a yellow highlight indicate an unexpected negative reaction*.

*Bovine str. = 3 bovine strains that gave identical results. These include 6013, ATCC19698 and 5077*.

*goat-bov = This column represents 1 goat strain and 1 bovine strain that gave identical results (4025 and 117p)*.

*2 strains = M. avium subs hominissuis strains 10-5894 and 10-05561, which had identical results*.

*6106/6007 = Two M. avium subspecies avium strains that gave identical results*.

#### Map

Eighty-six genes were confirmed as *Map*-specific by DNA amplification (Table 4). Conversely, four genes amplified non-*Map* DNA and thus are no longer considered *Map*-specific (Table 4). The primers designed from the MAP_RS01130-MAP_RS01135 combination amplified the correct size product in the *M. intracellulare* strain, but only faintly, perhaps indicating nucleotide mismatches in the primers. Primers to MAP_RS22390-MAP_RS00525 along with MAP_RS00515-MAP_RS00520 are all clustered together in the genome and amplified a correct sized product in two *Mah* strains (Table 4). MAP_RS08655 was the most conserved gene, amplifying a product in six of the nine non-*Map* strains tested (Table 4). Conversely, *Map* strain Linda failed to amplify seven genes, despite testing different *Taq* enzyme mixtures. A search of sequence read archive data from accession PRJEB2204 suggests all seven genes are present in the Linda strain, similar to all other *Map* strains. One primer pair designed from MAP_RS14095 failed to amplify DNA from any strain tested.

#### Non-map

Gene candidates that are diagnostic for *Mah* are very limited. Among the eight genes identified as specific to *Mah* by bioinformatic approaches, only three (FCV17_RS18630, FCV17_RS18640, and FCV17_RS18645) remained specific following PCR analysis as the other five genes were amplified a correct sized product from *M. intracellulare* DNA (Table 4). Of the eight *Maa*-specific genes selected for PCR analysis, seven were confirmed specific while only one (DBO90_RS13535) showed amplification in three *Map* strains. However, there are 149 other *Maa* gene candidates that were not tested, but could be *Maa*-specific (Supplementary Table 4).

The collective results identified 86 genes that are *Map*-specific, seven *Maa*-specific, and three *Mah*-specific. Thus, a final list of the subspecies-specific genes is included in Table 5. Collectively, these results further extended the number diagnostic targets tested for Johne's disease against 10 additional *Map* strains and 10 non-*Map* strains.

**Table 5:** *M. avium* subspecies-specific genes confirmed in this study.

**Original**	**RefSeq**			
**Locus tag**	**Locus tag**	**Protein ID**	**Description**	**Amino acids**
NA	MAP_RS00540	WP_003876878.1	XRE family transcriptional regulator	114
MAP0106c	MAP_RS00545	WP_003876023.1	Integrase	343
NA	MAP_RS04345	WP_016705558.1	Hypothetical protein	88
NA	MAP_RS04350	WP_003877462.1	Hypothetical protein	86
NA	MAP_RS04355	WP_003877465.1	Hypothetical protein	173
NA	MAP_RS04705	WP_003877504.1	Hypothetical protein	99
MAP2148	MAP_RS10920	WP_003878324.1	Recombinase XerD	227
MAP2151	MAP_RS10940	WP_003878325.1	Hypothetical protein	145
NA	MAP_RS10945	WP_019305592.1	Hypothetical protein	174
MAP2158	MAP_RS10965	WP_010949499.1	Hypothetical protein	193
MAP2179	MAP_RS11080	WP_003878349.1	Hypothetical protein	121
MAP2180c	MAP_RS11085	WP_016706156.1	Hypothetical protein	110
MAP2181c	MAP_RS11090	WP_003878351.1	TetR/AcrR family transcriptional regulator	206
MAP2182c	MAP_RS11095	WP_019305660.1	Nitroreductase family deazaflavin-dependent oxidoreductase	133
MAP2183c	MAP_RS11100	WP_003878354.1	Cytochrome P450	416
MAP2184c	MAP_RS11105	WP_003875947.1	NAD(P)-dependent oxidoreductase	261
MAP2189	MAP_RS11130	WP_003875942.1	MCE family protein	418
MAP2190	MAP_RS11135	WP_003875941.1	MCE family protein	341
MAP2191	MAP_RS11140	WP_003875940.1	MCE family protein	354
MAP2195	MAP_RS11160	WP_010949512.1	Hypothetical protein	448
NA	MAP_RS11165	WP_019305659.1	Hypothetical protein	72
NA	MAP_RS11170	WP_019305658.1	Hypothetical protein	159
MAP2473	MAP_RS12615	WP_003877285.1	Hypothetical protein	145
MAP2754	MAP_RS14055	WP_003875312.1	Hypothetical protein	85
MAP2755	MAP_RS14060	WP_003875311.1	Hypothetical protein	100
MAP2757	MAP_RS14070	WP_003875309.1	Hypothetical protein	79
NA	MAP_RS14110	WP_003878582.1	Hypothetical protein	92
MAP2763c	MAP_RS14120	WP_003878583.1	Hypothetical protein	74
MAP2765c	MAP_RS14130	WP_016706030.1	Hypothetical protein	296
NA	MAP_RS14135	WP_016706028.1	Helix-turn-helix domain-containing protein	70
MAP2767c	MAP_RS14140	WP_003875297.1	Hypothetical protein	183
MAP3436c	MAP_RS17665	WP_003878992.1	Hypothetical protein	231
NA	MAP_RS17670	WP_016705884.1	XRE family transcriptional regulator	87
MAP3726	MAP_RS19105	WP_019305717.1	Iron ABC transporter permease	324
MAP3727	MAP_RS19110	WP_003876873.1	ABC transporter ATP-binding protein	271
MAP3728	MAP_RS19115	WP_003873998.1	Fe^3+^-citrate ABC transporter substrate-binding protein	345
MAP3729	MAP_RS19120	WP_003873995.1	TauD/TfdA family dioxygenase	258
MAP3731c	MAP_RS19130	WP_010950082.1	Cobalt ABC transporter	500
MAP3732c	MAP_RS19135	WP_003873992.1	Energy-coupling factor transporter transmembrane protein EcfT	230
MAP3733c	MAP_RS19140	WP_003873991.1	Membrane protein	208
MAP3734c	MAP_RS19145	WP_003876870.1	ABC transporter ATP-binding protein	531
MAP3735c	MAP_RS19150	WP_019305718.1	ABC transporter ATP-binding protein	468
MAP3737	MAP_RS19155	WP_010950084.1	PPE family protein	504
MAP3738c	MAP_RS19160	WP_003876866.1	Class I SAM-dependent methyltransferase	244
MAP3740	MAP_RS19170	WP_010950086.1	Non-ribosomal peptide synthetase	3,068
MAP3743	MAP_RS19185	WP_019305720.1	Hypothetical protein	343
MAP3744	MAP_RS19190	WP_003873982.1	Thiazolinyl imide reductase	344
MAP3745	MAP_RS19195	WP_010950089.1	Thioesterase	250
MAP3746	MAP_RS19200	WP_003873980.1	Metal-sensitive transcriptional regulator	104
MAP3747c	MAP_RS19205	WP_010950090.1	Hypothetical protein	406
MAP3749	MAP_RS19215	WP_003873977.1	NAD(P)-dependent oxidoreductase	286
MAP3750	MAP_RS19220	WP_019305722.1	Transport accessory protein MmpS	140
MAP3751	MAP_RS19225	WP_010950093.1	MMPL family transporter	979
MAP3752	MAP_RS19230	WP_010950094.1	Acyl-CoA synthetase	578
NA	MAP_RS19240	WP_003876850.1	Hypothetical protein	131
MAP3756c	MAP_RS19245	WP_003876849.1	LLM class F420-dependent oxidoreductase	286
MAP3758c	MAP_RS19255	WP_019684230.1	AraC family transcriptional regulator	240
MAP3760c	MAP_RS19265	WP_003879162.1	Class I SAM-dependent methyltransferase	128
MAP3761c	MAP_RS19270	WP_003879163.1	GAP family protein	242
MAP3762c	MAP_RS19275	WP_003873965.1	Glycosyltransferase	408
MAP3763c	MAP_RS19280	WP_016705775.1	Acyltransferase	498
MAP3767c	MAP_RS19305	WP_003873957.1	30S ribosomal protein S18	88
MAP3768c	MAP_RS19310	WP_003873956.1	30S ribosomal protein S14	101
MAP3771	MAP_RS19325	WP_003873953.1	50S ribosomal protein L31 type B	97
NA	MAP_RS19330	WP_003879173.1	ANTAR domain-containing protein	87
MAP3772c	MAP_RS19335	WP_003873951.1	GTP-binding protein	380
MAP3774c	MAP_RS19345	WP_003879175.1	Metal ABC transporter permease	285
MAP3775c	MAP_RS19350	WP_003873948.1	ABC transporter	262
MAP3776c	MAP_RS19355	WP_019305700.1	ABC transporter substrate-binding protein	263
MAP3815	MAP_RS19565	WP_019305842.1	Hypothetical protein	254
NA	MAP_RS19570	WP_003873898.1	Hypothetical protein	49
MAP3817c	MAP_RS19575	WP_019305841.1	Carotenoid biosynthesis protein	278
MAP3818	MAP_RS19580	WP_016705755.1	Cytochrome P450	397
NA	MAP_RS22425	WP_023877748.1	Hypothetical protein	158
MAP0853	MAP_RS22505	WP_010948992.1	Hypothetical protein	219
MAP0856c	MAP_RS22515	WP_003872872.1	Hypothetical protein	575
MAP0860c	MAP_RS22520	WP_003872868.1	Hypothetical protein	296
MAP0862	MAP_RS22525	WP_003877468.1	Hypothetical protein	360
MAP0865	MAP_RS22535	WP_010949001.1	Cell division protein FtsK	423
NA	MAP_RS22540	WP_064469008.1	DNA-binding protein	75
MAP1610	MAP_RS22625	WP_010949277.1	Hypothetical protein	194
MAP2152c	MAP_RS22710	WP_010949495.1	Hypothetical protein	124
NA	MAP_RS22805	WP_073578986.1	Hypothetical protein	104
MAP2766c	MAP_RS22810	WP_003878585.1	DUF2742 domain-containing protein	158
MAP3730	MAP_RS22985	WP_003876872.1	Class I SAM-dependent methyltransferase	210
MAP3773c	MAP_RS23005	WP_003873950.1	Transcriptional repressor	139
	FCV17_RS18600	WP_084055242.1	Oxidoreductase	283
	FCV17_RS18640	WP_137987598.1	TetR/AcrR family transcriptional regulator	222
	FCV17_RS18645	WP_062890787.1	EVE domain-containing protein	138
	DBO90_RS12280	WP_009976179.1	Dihydrodipicolinate reductase	361
	DBO90_RS12340	WP_023879882.1	Hypothetical protein	134
	DBO90_RS12410	WP_009976156.1	Amidohydrolase	394
	DBO90_RS12500	WP_009976134.1	Zn-ribbon domain-containing OB-fold protein	137
	DBO90_RS12630	WP_009976097.1	SDR family oxidoreductase	267
	DBO90_RS18520	WP_009974923.1	IS110 family transposase	401
	DBO90_RS12105	WP_009976221.1	Cupin domain-containing protein	136

*“NA” indicates that gene is either not present or changed from the initial annotation, hence there is no original locus tag*.

*RefSeq locus tag prefix DBO90 is based on Maa HJW*.

*RefSeq locus tag prefix FCV17 is based on Mah 101174*.

#### A Single Reaction PCR Distinguishes All *M. avium* Subspecies

**Figure 3:**
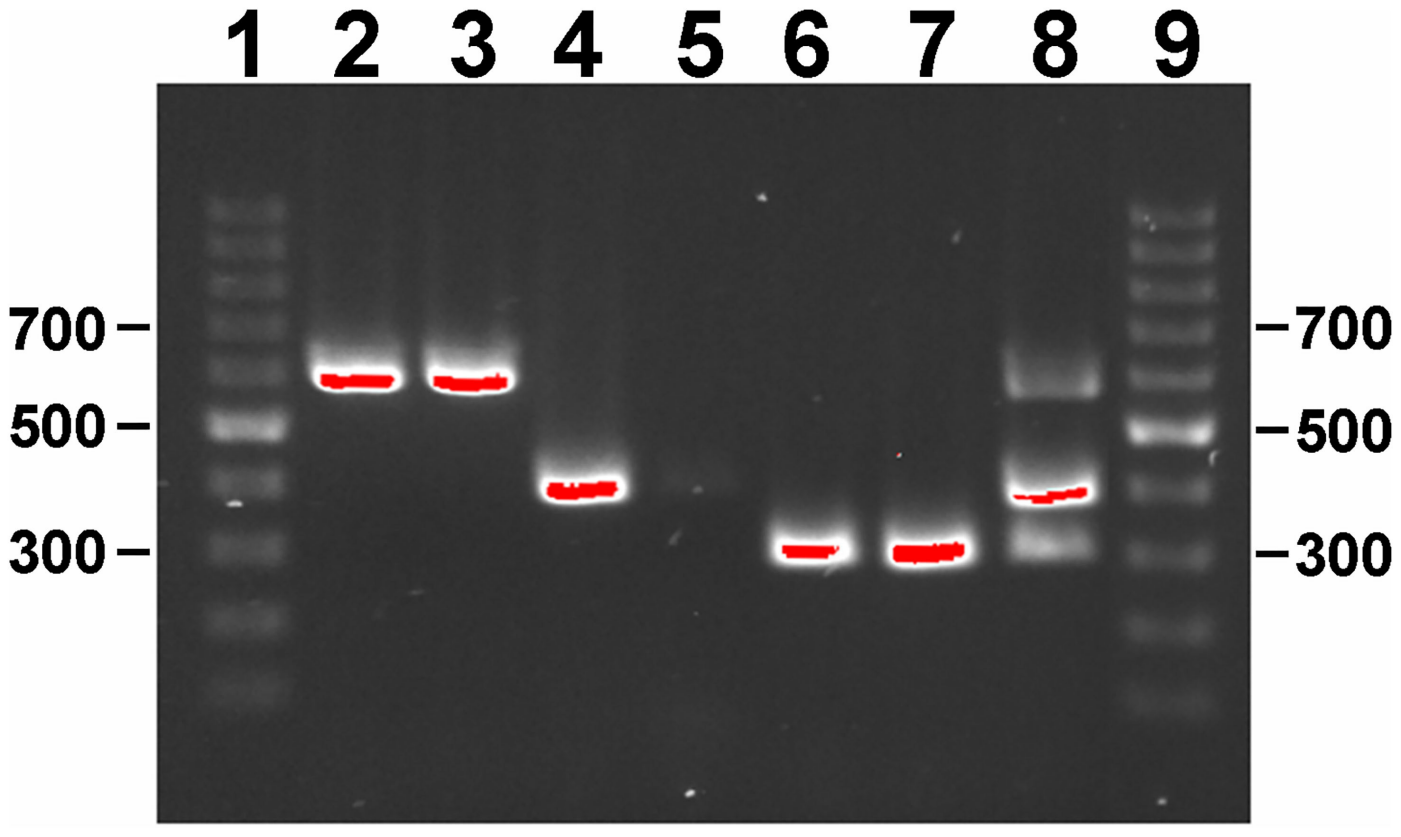
Gel electrophoresis of single-reaction PCR products on a 1% agarose gel stained with ethidium bromide. Lane assignments: 1 and 9 = 100 bp ladder; 2 = *Map* K-10; 3 = *Map* ATCC 19698; 4 = *Maa* Strain 18; 5 = *Maa* ATCC 35719; 6 = *Mah* HS 09-5894; 7 = *Mah* HS 10-1725; 8 = 117p-6084-MA 1115 mixture. Size standards are indicated in the left and right margins. Red indicates DNA band saturation.

As a proof of concept, primer combinations were tested that can distinguish any *M. avium* subspecies using a single-tube amplification reaction. Although other PCR reactions have been developed to distinguish *M. avium* subspecies (31), this approach eliminates the need for three reactions to accomplish the same result. This primer combination was used on selected genomic DNAs from *M. avium* subspecies. The single DNA amplification was able to distinguish each subspecies on the basis of size by gel electrophoresis (Figure 3), but can also be adapted to real time PCR using different Taqman probes and concentrations (32).

## Discussion

A total of 32 *Map*-specific genes were initially identified by comparing *Map* K-10 with *Mah* strain 104 (17, 20, 22). These genes have aided the specific detection of *Map*, but are single-copy genes compared to IS900, a repetitive element that was known to be diagnostic for *Map* long before whole-genome comparisons were performed (9, 10). Using a comparative genomic approach combined with PCR analysis, this study confirmed 18 of the original 32 genes and identified 68 additional *Map*-specific genes as diagnostic targets (Table 5) yielding a total of 86 genes. Some genes were initially identified as potentially subspecies specific after PanOCT and Roary analysis, but were subsequently found to be conserved in other mycobacteria after additional strains were tested by *in silico* PCR or *Taq*-based PCR analysis. For example, eight *Mah*-specific genes were identified by bioinformatic analysis, but only three were still deemed *Mah*-specific following DNA amplification experiments. The number of subspecies-specific genes will likely be further reduced as additional *Map* and non-*Map* strains are analyzed. Significantly more genes specific to *Map* vs. *Mah* are likely attributed to the closed, stable genome of *Map*, which increases the chance that specific genes are present in all strains. The opposite is true for *Mah*, which has a more dynamic, open genome containing a smaller number of core sequences (2) and therefore, the number of specific genes is very small. *Maa* had the highest number of subspecies specific genes, possibly because only two complete genome sequences were bioinformatically analyzed for members of this subspecies. Only eight of the 157 *Maa* genes were selected for DNA amplification studies and seven genes remained specific to *Maa*. Nonetheless, with these novel data, researchers can classify isolates in their freezers, quickly characterize clinical samples, and functionally analyze these unique genes.

A total of 40% of the *Map*-specific genes and 24% of *Maa*-specific genes are annotated as hypothetical. This large percentage of *Map* hypothetical proteins is interesting considering the updated RefSeq annotation has resulted in a significant drop in the overall number of hypothetical proteins, but it suggests how little is known about these novel subspecies-specific genes. Also notable among this group is an operon of genes encoding Mce family proteins (Table 5), some of which are surface exposed and play a role in virulence and lipid transport (33). These proteins are typically conserved among the mycobacteria, and at least one gene this group is no different as *in silico* PCR analysis showed that the correct sized amplification product is obtained using MAP_RS11145 primers on *M. marseillence* strains (Supplementary Table 6). This species of *Mycobacterium* is considered a member of the *Mycobacterium avium* complex based on 16S rRNA, *rpoB*, and *hsp65* sequence similarity (25).

There are significant differences between PanOCT and Roary analysis in terms of genes assigned to subspecies-specific orthologous clusters. There are 41 genes identified as *Map*-specific by Roary that are not considered specific by PanOCT analysis. These discrepancies are attributed to differences in the algorithms of these two bioinformatic tools. PanOCT uses a single clustering tool compared to Roary, which uses two clustering mechanisms and then merges the results. In addition, Roary appears more stringent in the gene cluster formation through the use of BLASTP identity thresholds of 95%, which yields more gene clusters. This may explain why the Roary output listed more gene clusters specific to *Map* than PanOCT. Roary stringency results in more gene clusters overall since Roary will not cluster genes less identical than the threshold. Thus, Roary will generate more groups of subspecies-specific genes than PanOCT causing Roary to estimate a higher number of subspecies-specific genes. On the other hand, PanOCT will group some of the genes because of its lower stringency and the number of specific groups will be lower.

Other discrepancies not related to mechanistic processes used by each tool can be traced to unexpected annotation differences in very similar orthologs. It is important to note that PanOCT is designed to give context to its findings in relation to genome organization and works off annotation data. This may be the reason why there is only one *Map*-specific gene identified by PanOCT analysis that is not listed by Roary. That one gene, encoding alpha/beta hydrolase (MAP_RS19250 in K-10), has the same translational start for all of the *Map* strains except Telford, which starts 95 codons downstream of the other strains (Supplementary Figure 1). This prevented its inclusion by Roary, and thus it was not considered a *Map*-specific gene, but it is indeed *Map*-specific. The same situation can be applied to the *Mah*-specific gene (MAV_RS08635 in strain 104), which encodes hydroxycinnamic acid hydroxylase (Table 3). These results pose the question of which bioinformatic approach works better for defining these diagnostic gene targets. Since agreement of both pipelines was required, the more conservative method would drive the final list of targets. Is the conservative assessment by PanOCT too strict or is Roary too liberal? Or is it somewhere in between? The only way to answer the question of whether the conservative nature of PanOCT was warranted would be to test those genes identified only by Roary to determine their diagnostic specificity. When the 41-gene subset from Roary analysis was further analyzed by BLAST against the NCBI database, most of these genes (31 of 41) showed reasonably strong homologies to other, more distantly related, *Mycobacterium* species (Supplementary Table 3). Therefore, PanOCT might be the better bioinformatic tool to use in this approach.

There are several possible reasons for the non-specific amplification of selected genes that were reported as specific by PanOCT and Roary analysis. First, the region amplified in non-*Map* strains may not have been called as a protein coding region (pseudogene, intergenic region, or frameshifted protein). Second, the region amplified in non-*Map* strains may be in a protein coding region that is annotated in a different reading frame compared to *Map* strains. Third, underlying errors in genomic sequence could produce both these types of annotation deviations or the regions may be near a site where one of the strain clusters is reorganized compared to the other cluster. One example is MAP_RS22905, a gene specific to *Map* based on PanOCT and Roary, but amplifies a product in non-*Map* strains by *in silico* PCR and Taq-based PCR. This gene encodes a hypothetical protein and is immediately upstream of MAP_RS17350 in *Map* K-10, but with a significant 27-amino acid overlap in these coding sequences. MAP_RS17350 is annotated as a frameshifted non-functional protein that contains several internal stop codons. The primers to this gene were designed such that the forward primer binds within the MAP_RS22905 coding sequence while the reverse primer binds within the coding sequence that overlaps with MAP_RS17350. In the non-*Map* strains (as well as other *Map* strains) there is a single protein that is functional and encompasses the sequence that corresponds to MAP_RS22905 and MAP_RS17350. Therefore, it is important to examine the location of predicted amplicons in the genome context to clear up some ambiguities.

While some *Taq*-based amplification reactions were unexpectedly positive, and thus non-specific, other reactions were unexpectedly negative. One *Map*-specific gene (MAP_RS14095) did not amplify any of the genomic DNAs using the primer pairs (Table 4). These primers were carefully selected using strict parameters which resulted in a PCR product of the correct size by *in silico* PCR, but still did not amplify a product using purified genomic DNAs. Therefore, this primer pair was considered bad and MAP_RS14095 has yet to be confirmed as subspecies-specific. An additional six *Map*-specific genes failed to amplify using only the *Map* strain Linda template, although all of the other genes were successfully amplified with this genomic DNA sample. It is unclear why these genes did not amplify with this one template, since a search of unfinished contigs for the Linda strain suggests these six genes are indeed present. Nonetheless, these genes were not included among the *Map*-specific genes because they didn't amplify using two independent commercial recipes.

The insertion sequence IS900 and many other repeat elements were not identified by PanOCT or Roary analysis. This is due to the lack of such elements included in the two *Map* Egyptian isolates E1 (CP010113.1) and E93 (CP010114.1) (34). Applying the criteria used in this study that the gene must be present in all *Map* and absent in all non-*Map*, these sequences do not appear in the E1 and E93 strains for reasons speculated previously (2), and hence they did not make the list. Nonetheless, there is no doubt that these two commonly used repetitive sequences are indeed *Map* specific and present in all *Map* strains. Surprisingly, the ISMap*02* sequence did show positive PCR reactions with some *Mah* strains, but not with *Maa* (data not shown), casting doubt on its diagnostic specificity. Repetitive sequences specific to *Mah* and *Maa* were also identified in this study. One example is DBO90_RS18520 (from *Maa* HJW), an IS110 family transposase that has 14–16 copies in *Maa* strains.

F57, *hspX*, and locus 251 have been widely used as diagnostic sequences for *Map* (11, 13, 18, 35). These genes were checked against the subspecies-specific gene lists obtained from the current analyses. The F57 sequence occurs within MAP_RS22535 (MAP_0865) and was also identified as *Map*-specific in this study. Likewise, *hspX* was originally discovered as a *Map*-specific gene before the whole genome sequence was available (11) and was subsequently shown to be expressed within *Map*-infected macrophages (12). The locus tag for this sequence is MAP_RS11095 (MAP_2182c) and has been used in studies to determine antigenic reactivity (36). Locus 251 has been tested in a real-time PCR platform and was considered *Map*-specific in that context (14). All three of these sequences were also identified in this study as *Map*-specific by Roary, PanOCT, and PCR analysis.

One area where annotations have improved dramatically has been in G+C rich organisms like the mycobacteria. RefSeq annotation has identified new coding sequences that were previously hidden in the same genome because original annotation software either missed them or called distinctly different start sites or reading frames for the same gene. Because of the consistency in the RefSeq annotation pipeline, genomes can now be directly compared at the gene feature level. Several subspecies specific genes identified in this study do not list an outdated locus tag, but only the updated RefSeq locus tag. Among the *Map*-specific genes, 17 do not have an original locus tag designation (Table 5), representing 20% of the *Map*-specific genes identified in this study that might have been “missed” or changed significantly from the initial sequence annotation (37). For example, closer inspection of MAP_RS04350, which does not list an original locus tag in Table 5 and is annotated as a hypothetical protein, actually encodes the C-terminal 86 amino acids of MAP_0852 which was previously shown to be *Map*-specific (22). Other similar examples of shifted coding sequences that result in dropped locus tags from the original annotation exist as well. In our previous studies, we expressed *Map*-specific coding sequences identified by the original annotation and tested them for antibody reactivity (38). Although specific antigens were identified, additional testing revealed low antibody reactivity to all *Map*-specific coding sequences known at that time. Now with these updated RefSeq coding sequences identified by the prokaryotic genome annotation pipeline (PGAP), there still remains a possibility to obtain a truly specific and strong antigen for *Map*. Therefore, these updated RefSeq coding sequences should be recombinantly expressed and examined for antigenicity as has been done with previous *Map*-specific genes (38).

## Data Availability Statement

The original contributions presented in the study are included in the article/Supplementary Materials, further inquiries can be directed to the corresponding author/s.

## Author Contributions

JB conceived and designed the study. All authors made substantial contributions to the experimentation, analysis, and writing of the manuscript.

## Conflict of Interest

The authors declare that the research was conducted in the absence of any commercial or financial relationships that could be construed as a potential conflict of interest.
