# Supplementary material for: Diagnostic Sequences That Distinguish M. avium Subspecies Strains
Source: Front Vet Sci. 2021 Jan 28;7:620094. doi: 10.3389/fvets.2020.620094 (PMC7876471; doi:10.3389/fvets.2020.620094)
Supplement: Supplementary file 7 [file Data_Sheet_1.docx]

Scripts used in this study.

import argparse

import numpy

import pandas

from GenomicPackage.genbank import Genome

def parse_argument():

parser = argparse.ArgumentParser()

parser.add_argument("-i", "--eggnog_result", type=str, required=True, help="")

parser.add_argument("-l", "--gene_list", type=str, required=True, help="List of locustag pass to the eggnog-mapper tool")

parser.add_argument("-d", "--cog_function_cat", type=str, default="fun-20.tab", help="")

parser.add_argument("-g", "--genbank", nargs="+", help="Genbank file to retrieve annotation of gene")

parser.add_argument("-o", "--output", type=str, required=True, help="Output File")

parsed_args = parser.parse_args()

return parsed_args

def get_annotation(locustag, genomes):

annotation = ""

for genome in genomes:

try:

annotation = genome[locustag].product

except KeyError:

continue

return annotation

else:

return numpy.nan

if __name__ == '__main__':

args = parse_argument()

eggnog_result = pandas.read_csv(args.eggnog_result, sep="\t", comment="#", index_col=False, names=['query_name', 'seed_eggNOG_ortholog', 'seed_ortholog_evalue', 'seed_ortholog_score', 'best_tax_level', 'Preferred_name', 'GOs', 'EC', 'KEGG_ko', 'KEGG_Pathway', 'KEGG_Module', 'KEGG_Reaction', 'KEGG_rclass', 'BRITE', 'KEGG_TC', 'CAZy', 'BiGG_Reaction', 'taxonomic_scope', 'eggNOG_OGs', 'best_eggNOG_OG', 'COG_Functional_cat', 'eggNOG_free_text_desc']).set_index("query_name")

cog_function_cat = pandas.read_csv(args.cog_function_cat, sep="\t", names=["COG_Functional_cat", "hex_color", "desc"]).set_index("COG_Functional_cat")

original_locustag_list = []

with open(args.gene_list) as list_in:

for locustag in list_in.read().splitlines():

original_locustag_list.append(locustag)

final_df = pandas.DataFrame()

columns = ['locustag', 'annotation', 'taxonomic_scope', 'best_tax_level', 'Preferred_name', 'seed_ortholog_evalue', 'KEGG_ko', 'EC', 'eggNOG_OGs', 'COG_color', 'COG_functional_cat_desc', 'eggNOG_free_text_desc']

genomes = [Genome(gbk) for gbk in args.genbank]

for locustag in original_locustag_list:

try:

row = eggnog_result.loc[locustag, :]

cog_cat = row.COG_Functional_cat

if not type(cog_cat) == str:

cog_cat = "S"

for cat in cog_cat:

data = [row.name, get_annotation(row.name, genomes), row.taxonomic_scope, row.best_tax_level, row.Preferred_name, row.seed_ortholog_evalue, row.KEGG_ko, row.EC, row.eggNOG_OGs, *cog_function_cat.loc[cat, :].to_list(), row.eggNOG_free_text_desc]

data = {i: v for i, v in enumerate(data)}

temp = pandas.DataFrame(data=data, index=[0])

temp.columns = columns

final_df = final_df.append(temp, ignore_index=True, sort=False)

except KeyError:

data = [locustag, get_annotation(locustag, genomes), *[numpy.nan] * 8, "No result", numpy.nan]

data = {i: v for i, v in enumerate(data)}

temp = pandas.DataFrame(data=data, index=[0])

temp.columns = columns

final_df = final_df.append(temp, ignore_index=True, sort=False)

final_df.to_csv(args.output, sep="\t", index=False)
