# Supplementary material for: Diagnostic Sequences That Distinguish M. avium Subspecies Strains
Source: Front Vet Sci. 2021 Jan 28;7:620094. doi: 10.3389/fvets.2020.620094 (PMC7876471; doi:10.3389/fvets.2020.620094)
Supplement: Supplementary file 8 [file Data_Sheet_2.docx]

Scripts used in this study.

import json

import os

import argparse

import numpy

import pandas

import itertools

import matplotlib.pyplot as plt

import seaborn as sns

sns.set()

from GenomicPackage.genbank import Genome

def parse_arguments():

parser = argparse.ArgumentParser()

parser.add_argument("-d", "--json_database", type=str, required=True, help="KEGG database link K to ko")

parser.add_argument("-f", "--kegg_results", type=str, required=True, help="Raw result file from kofamscan")

parser.add_argument("-o", "--output", type=str, required=True, help="Output File")

parser.add_argument("-g", "--genbank", nargs="*", help="Genbank file to retrieve annotation of gene")

parser.add_argument("-e", "--evalue", type=float, default=1e-10, help="Evalue threshold used to assign function class to record without a score ratio. [1e-10]")

parser.add_argument("-c", "--funcat", type=str, default="", help="Tab file containing a list of functional categories and associated color")

parsed_args = parser.parse_args()

return parsed_args

def get_annotation(locustag, genomes):

annotation = ""

for genome in genomes:

try:

annotation = genome[locustag].product

except KeyError:

continue

return annotation

def get_ratio(row):

thres = row["threshold"]

score = row["score"]

ratio = round((score / thres) * 100, 2)

return ratio

def parse_KEGG_level(row):

lvl1 = row["KEGG Level 1"]

if "Brite Hierarchies" in lvl1:

lvl2 = row["KEGG Level 2"].split(":")[1].strip().title()

return lvl2

elif "Not Included in Pathway or Brite" in lvl1:

lvl2 = row["KEGG Level 2"]

if "Unclassified" in lvl2:

lvl2 = lvl2.split(":")[1].strip().title()

return lvl2

else:

return lvl2.capitalize()

elif "Environmental Information Processing" in lvl1:

return lvl1

elif "Genetic Information Processing" in lvl1:

return lvl1

elif "Human Diseases" in lvl1:

return lvl1

elif "NA" in lvl1:

return "Unknown Function"

else:

lvl2 = row["KEGG Level 2"].title()

return lvl2

def get_functional_class(row, threshold):

ratio = row["ratio"]

if not numpy.isnan(ratio):

if ratio >= 50:

result = parse_KEGG_level(row)

return result

else:

return "Unknown Function"

else:

# check evalue

evalue = float(row["evalue"])

if evalue < threshold:

result = parse_KEGG_level(row)

return result

else:

return "Unknown Function"

def get_colors(x, fun_cat):

color = fun_cat.loc[x].values[0]

rgb = convert_color(color)

return rgb

def convert_color(hex_code):

iter_hex_code = iter(hex_code)

rgb = [int("".join(itertools.islice(iter_hex_code, 2)), 16) / 255 for i in range(len(hex_code) // 2)]

return rgb

if __name__ == '__main__':

args = parse_arguments()

with open(args.json_database) as in_database:

database = json.load(in_database)

raw_df = pandas.read_csv(args.kegg_results, sep="\t", comment="#", names=["confidance", "locustag", "K", "threshold", "score", "evalue", "definition"])

raw_df["ratio"] = raw_df.loc[:, ["threshold", "score"]].apply(get_ratio, axis=1)

parsed_df = pandas.DataFrame(columns=raw_df.columns)

groups = raw_df.groupby("locustag")

for name, group in groups:

# try to find ratio >= 100

ratio = group[group["ratio"] >= 100]

if not ratio.empty:

parsed_df = parsed_df.append(ratio.iloc[0])

continue

# No ratio found >= 100 then sort by evalue and take the first result

sorted_group = group.sort_values("evalue")

parsed_df = parsed_df.append(sorted_group.iloc[0])

pre_final_df = parsed_df.loc[:, ["confidance", "locustag", "K", "ratio", "evalue"]]

if args.genbank:

genomes = [Genome(gbk) for gbk in args.genbank]

pre_final_df["Annotation"] = pre_final_df["locustag"].apply(get_annotation, args=(genomes,))

final_df = pandas.DataFrame()

columns = list(pre_final_df.columns) +["KEGG Level 1", "KEGG Level 2", "KEGG Level 3", "KEGG Level 4"]

for row in pre_final_df.itertuples():

try:

search_result = database[row.K]

except KeyError:

data = list(row[1:]) + ["NA"] * 3 + parsed_df[(parsed_df["K"] == row.K) & (parsed_df["locustag"] == row.locustag)]["definition"].to_list()

data = {i: v for i, v in enumerate(data)}

temp = pandas.DataFrame(data=data, index=[0])

temp.columns = columns

final_df = final_df.append(temp, ignore_index=True, sort=False)

continue

for h in search_result:

data = list(row[1:]) + h

data = {i: v for i, v in enumerate(data)}

temp = pandas.DataFrame(data=data, index=[0])

temp.columns = columns

final_df = final_df.append(temp, ignore_index=True, sort=False)

for col in final_df.columns[6:]:

final_df[col] = final_df[col].apply(lambda x: " ".join(x.split()[1:]) if x else "NA")

# Functional Assignation

final_df["Function Class"] = final_df.apply(get_functional_class, args=(args.evalue, ), axis=1)

if args.funcat:

fun_cat = pandas.read_csv(args.funcat, sep="\t", names=["color", "Functional cat"]).set_index("Functional cat")

final_df["color"] = final_df["Function Class"].apply(get_colors, args=(fun_cat, ))

final_df.to_csv(args.output, sep="\t", index=False)

# Plot Pie chart

if args.funcat:

counter = final_df.groupby("Function Class").size()

pie, ax = plt.subplots(figsize=[12,6])

labels = counter.keys()

colors = [convert_color(fun_cat.loc[label].values[0]) for label in labels]

plt.pie(x=counter, autopct="%.1f%%", explode=[0.05]*len(counter), colors=colors, labels=labels, pctdistance=0.8)

plt.savefig(os.path.splitext(args.output)[0] + ".png", dpi=300)
