# Supplementary figures and images for: Diagnostic Sequences That Distinguish M. avium Subspecies Strains
Source: Front Vet Sci. 2021 Jan 28;7:620094. doi: 10.3389/fvets.2020.620094 (PMC7876471; doi:10.3389/fvets.2020.620094)

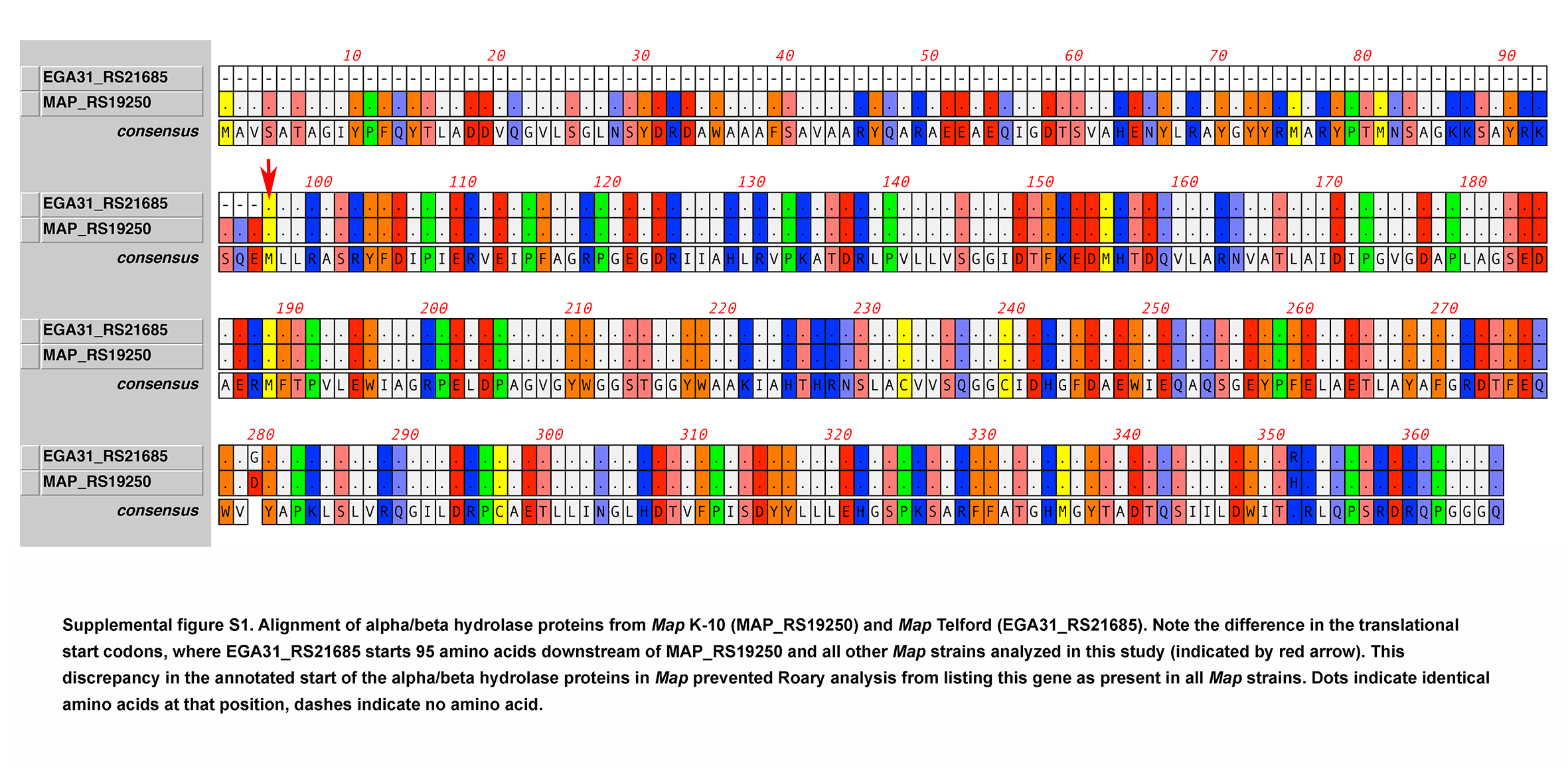

Supplement: Supplementary file 9 [file Image_1.TIF]
